# Thirty-Day and One-Year Outcomes of Endovascular Treatments for Severe Atherosclerotic Stenosis of Intracranial ICA: Results From a Single Center

**DOI:** 10.3389/fneur.2021.668868

**Published:** 2021-05-25

**Authors:** Shengli Shen, Yingjin Wang, Xudong He, Ning Ma, Feng Gao, Ligang Song, Xuan Sun, Lian Liu, Zhongrong Miao, Hongzhou Duan, Dapeng Mo

**Affiliations:** ^1^Department of Neurosurgery, Peking University First Hospital, Beijing, China; ^2^Department of Neurology, School of Medicine, Sir Run Run Shaw Hospital, Zhejiang University, Hangzhou, China; ^3^Department of Interventional Neuroradiology, Beijing Tiantan Hospital, Capital Medical University, Beijing, China

**Keywords:** endovascular treatment, atherosclerotic stenosis, intracranial internal carotid artery, outcome, complication

## Abstract

**Background:** Endovascular treatment for intracranial atherosclerotic stenosis (ICAS) has been developed. However, the intracranial internal carotid artery (ICA) presents a particular challenge due to the location and tortuous route, and the outcomes of endovascular treatment in patients with stenosis of the intracranial ICA still have not been reported. This article retrospectively investigated the 30-day and 1-year outcomes of tailored endovascular treatment for patients with severe intracranial ICA stenosis from a single center.

**Methods:** Between June 2014 and December 2017, 96 consecutive patients with severe atherosclerotic stenosis (70–99%) of the intracranial ICA were managed with endovascular treatment in Beijing Tiantan Hospital. Three different kinds of treatments [angioplasty with balloon dilatation alone (BD group), balloon-mounted stent (BMS group), and self-expanding stent (SES group)] were performed according to the characteristics of the lesions. The primary endpoints included any stroke or death within 30 days and ipsilateral ischemic stroke afterwards within 1 year. Secondary endpoints included the revascularization success rate (residual stenosis <30%) and the restenosis rate (stenosis ≥ 50%) within 1 year.

**Results:** The 30-day death rate was 0, and the stroke rate of all patients was 7.3% (7/96). The stroke rate was higher in the BD group (15.8%) and SES group (9.8%) than in the BMS group (0%) (*p* = 0.047). Thirteen (13.5%) patients suffered at least one onset of ischemic stroke in the ipsilateral ICA territory within 1 year, and there was no significant difference among the three groups (*p* = 0.165). The overall revascularization success rate was 93.8%, and the revascularization success rate was significantly higher in the SES group (100%) than in the BD group (78.9%) (*p* = 0.006). The restenosis rate of all patients within 12 months was 20.8%, and there was no significant difference among the three groups. Patients with Mori type C target lesions were more likely to suffer stroke within 30 days (25%) and restenosis within 1 year (31.3%).

**Conclusions:** Both the 30-day and 1-year outcomes of tailored endovascular treatments seemed to be acceptable in the treatment of symptomatic atherosclerotic stenosis of the intracranial ICA. However, this needs to be confirmed by further investigation, preferably in large multicenter randomized controlled clinical trials.

## Introduction

Intracranial atherosclerotic stenosis (ICAS) is a common etiology of stroke worldwide, with the highest prevalence being in Asian, Hispanic, and African populations and ICAS accounting for approximately half of transient ischemic attacks (TIAs) and ischemic strokes in Asian populations (1, 2). The annual risk of recurrent stroke in patients with ICAS varies from 4 to 40% and is especially high in patients with severe stenosis (70–99%), for which the rate is ~23% despite best medical therapy (BMT) (3, 4). Endovascular treatments, including percutaneous transluminal angioplasty (PTA) with balloon dilatation (BD) alone and PTA with stenting (PTAS) using either a balloon-mounted stent (BMS) or a self-expanding stent (SES) have been developed and widely used in some countries. These treatments initially appeared to be safe and effective (5). However, two randomized controlled trials [Stenting and Aggressive Medical Management Therapy for Preventing Recurrent Stroke in Intracranial Stenosis (SAMMPRIS) and Vitesse Intracranial Stent Study for Ischemic Therapy (VISSIT)] did not show superiority of stenting over aggressive medical management alone for ICAS, with a high incidence of periprocedural complications and recurrent stroke (6, 7). Restenosis is another major drawback with a rate of up to 34% (8).

One of the major concerns about the SAMMPRIS and VISSIT trials is that only one type of stent was used in each trial. However, different lesions are suitable for different treatments, and selecting either a BMS or SES based on the characterization of lesions might achieve a better clinical result (3, 9). In addition, the clinical outcome of endovascular treatment is also associated with the location of arteries (10). Symptomatic intracranial internal carotid artery (ICA) atherosclerotic stenosis plays an important role in ischemic stroke and responds poorly to anticoagulation therapy (11). Due to the location and tortuous route of the intracranial ICA, endovascular treatment for stenosis of intracranial ICA presents a particular challenge and is relatively underdeveloped, with more perioperative complications, higher long-term in-stent restenosis rates, and higher recurrent rates of ischemic stroke (12, 13). Currently, clinical trials with 30-day outcomes of tailored endovascular treatment for ICAS and endovascular treatments for intracranial ICA stenosis with small samples have been reported, while outcomes of individualized tailored endovascular treatments for intracranial ICA stenosis with large samples and a longer follow-up have not been reported (11, 14). This article aimed to investigate the 30-day and 1-year outcomes of tailored endovascular treatments for severe intracranial ICA stenosis.

## Materials and Methods

### Patients and Study Design

This retrospective study was carried out at a tertiary stroke center and approved by the institutional ethics committee at Beijing Tiantan Hospital. Between June 2014 and December 2017, 96 consecutive patients with severe atherosclerotic stenosis of the intracranial ICA (70–99%), including the C5, C6, and C7 segments of the ICA were managed with endovascular treatment. The identification of segments of ICA was performed according to Bouthillier's classification system (15). Inclusion criteria were as follows: (1) Patients over 18 years old; (2) TIA or stroke attributable to the severe stenosis (≥70%) of intracranial ICA within the past 30 days; (3) stenosis was verified by digital subtraction angiography (DSA) and measured according to the warfarin-aspirin symptomatic intracranial disease (WASID) trial criteria with normal distal vessels as the reference; (4) ipsilateral hypoperfusion (≥40% decrease in cerebral blood flow in the territory distal to the target lesion) with poor collaterals by CT perfusion; and (5) atherosclerotic stenosis and having at least one atherosclerotic risk factor (hypertension, diabetes mellitus, hyperlipidemia, hyperhomocysteinemia, and smoking) (4, 14, 16). Exclusion criteria included (1) non-atherosclerotic causes of intracranial stenosis (arterial dissection, vasculitis, etc.), potential source of cardiac embolism, or concurrent intracranial pathology such as Moyamoya disease; (2) concurrent intracranial tumor, cerebral aneurysm, or arteriovenous malformation; (3) known contraindication to aspirin, clopidogrel, heparin, tirofiban, contrast media, metal, etc.; (4) life expectancy <1 year because of other medical conditions such as cancer; and (5) other conditions not suitable for general anesthesia or endovascular treatment.

### Strategy of Endovascular Treatment

DSA was performed for all patients, and the strategy of endovascular treatment was decided according to the site and characteristics of the target lesions and based on the operators' experience and preference (3, 9). In general, Apollo BMS (MicroPort Medical, Shanghai, China) was preferred in lesions with straightforward arterial access. SES (Wingspan, Boston Scientific; Enterprise, Codman Neurovascular; Solitaire, Covidien; Neuroform, Smart Therapeutics/Boston Scientific) with balloon pre-dilatation were selected in lesions with tortuous arterial access or lesions with a significant mismatch in the diameter between the proximal and distal segments. BD alone (Gateway, Stryker Neurovascular, USA) was performed in patients with more tortuous arterial access for which stenting was considered to be improper by operators (17).

### Periprocedural Management

Aggressive medical management and intensive risk factor management were implemented pre-procedurally, including aspirin (100 mg/day), clopidogrel (75 mg/day), atorvastatin calcium (20 mg Qn), platelet function monitoring by thromboelastography, strict blood glucose control, and cigarette control.

Endovascular treatments were performed by experienced neurointerventionists. Either general anesthesia or local anesthesia was chosen based on the operator's experience and the patient's condition, and most of the procedures were performed under general anesthesia. An intravenous heparin regimen was administered to maintain an activated clotting time between 250 and 300 s during the procedure. Briefly, a 6F-guiding catheter was introduced through the common femoral artery and guided into the target ICA. After a microwire was first passed through the intracranial stenosis, one of the three kinds of interventional procedures (BD, BMS, SEM) was performed based on the operators' experience. The devices of endovascular treatment were as follows: BD alone with Gateway balloon, PTAS with the Apollo BMS, or balloon predilatation and subsequent deployment of a SES (Wingspan, Enterprise, Solitaire, or Neuroform).

Dual antiplatelet therapy was maintained for at least 6 months, and aspirin alone was continued daily afterwards. Rehabilitation treatment was recommended for patients with functional disability. Long-term management of individual medical risk factors such as blood pressure, cholesterol, and diabetes mellitus was implemented.

### Patient Follow-Up and Outcome Measures

All patients were followed up regularly. CT angiography or CT perfusion was performed at the 30-day or 6-month follow-up. DSA was performed in the 6-month or 1-year follow-up. MRI was recommended if the patient had symptoms of ischemia. The primary endpoints were any stroke or death within 30 days and ipsilateral ischemic stroke beyond 30 days after angioplasty. Recurrent ischemic stroke was considered to be any focal neurological symptom of sudden onset that lasted for at least 24 h, was related to the corresponding vascular territory, was not associated with a hemorrhage on brain CT or MRI, and occurred within the follow-up period (8). Secondary endpoints included the revascularization success rate and the restenosis rate at the 1-year follow-up. The revascularization success of endovascular treatment was based on the following criteria: (1) the blood flow was unobstructed, reaching TICI grade 3; (2) the residual stenosis was <30% (measured according to WASID trial criteria); (3) the distal vessels were not embolized or missing; and (4) there was no leakage of contrast medium. Restenosis was defined as 50% or greater diameter stenosis at the follow-up angiography.

### Statistical Analysis

The data were primarily analyzed by intention-to-treat analysis. Continuous variables were presented as the mean ± standard deviation (SD) or median with interquartile range (IQR), as appropriate. The differences between groups were determined by one-way analysis of variance or Kruskal-Wallis test. Categorical variables were expressed as percentages, for which the chi-square test or Fisher's exact tests were used. Binary logistic regression analysis was used to relate the occurrence of the endpoints to multiple clinical factors, and factors were included as candidates for inclusion in the model if the probability value for the bivariate association with the endpoint was <0.2. A two-tailed *p*-value < 0.05 was considered statistically significant. Statistical analysis was performed using SPSS® software (SPSS Statistical Software 26).

## Results

### Baseline Characteristics

Between June 2014 and December 2017, 124 consecutive patients with severe atherosclerotic stenosis (70–99%) of the C5-7 segments of the ICA were screened and managed with endovascular treatment in Beijing Tiantan Hospital, among which 96 patients (60.99 ± 8.00 years old) were followed up for at least 1 year. The 96 patients included 64 (66.7%) males and 32 (33.3%) females. According to the morphology and length of the stenosis lesions, different strategies of endovascular treatments were selected. Of the 96 patients, 19 (19.8%) were treated with BD alone (BD group), 36 (37.5%) were treated with BMS (BMS group), and 41 (42.7%) were treated with SES (SES group).

The baseline characteristics of the patients are presented in Table 1. The median mRS score of all enrolled patients was 0 (IQR 0–1), and the National Institutes of Health Stroke Scale (NIHSS) score was 0 (IQR 0–2). The most common risk factor was hypertension (75.0%), followed by diabetes mellitus (53.3%), and hyperlipidemia 35.4%. A total of 44.8% of patients had a past history of smoking or were still smoking. A total of 9.4 and 2.1% of patients had a past history of myocardial infarction and atrial fibrillation, respectively. There was no significant difference in functional scale, common risk factors, or relative disease history between the three groups.

**Table 1:** Baseline characteristics of patients receiving endovascular treatment in different groups.

	**Balloon**	**Balloon-mounted**	**Self-expanding**	**Total**	***p*-value**
	**dilatation**	**stent**	**stent**		
*N* (%)	19 (19.8)	36 (37.5)	41 (42.7)	96	
Sex: male (%)	12 (63.2)	31 (86.1)	21 (51.2)	64 (66.7)	0.005
Age in years [mean (SD)]	63.9 (8.4)	60.3 (8.0)	60.3 (7.8)	61.0 (8.0)	0.211
BMI [mean (SD), kg/m^2^]	26.8 (7.31)	24.4 (1.76)	25.5 (2.9)	25.3 (3.8)	0.112
mRS [median (IQR)]	0 (0, 2)	0 (0, 1)	0 (0, 1)	0 (0, 1)	0.934
NIHSS [median (IQR)]	0 (0, 3)	0 (0, 2)	0 (0, 1)	0 (0, 2)	0.376
Hypertension (%)	14 (73.7)	27 (75.0)	31 (75.6)	72 (75.0)	0.979
Hyperlipidemia (%)	7 (36.8)	11 (30.6)	16 (39.0)	34 (35.4)	0.733
Diabetes mellitus (%)	11 (57.9)	12 (33.3)	26 (63.4)	49 (53.3)	0.046
Smoking (%)	11 (57.9)	22 (61.1)	10 (24.4)	43 (44.8)	0.018
Coronary disease (%)	2 (10.5)	5 (13.9)	2 (4.9)	9 (9.4)	0.389
Atrial fibrillation (%)	0 (0.0)	1 (2.8)	1 (2.4)	2 (2.1)	1.000
Stroke history (%)	0 (0.0)	1 (2.8)	2 (4.9)	3 (3.1)	1.000
Stenosis [%, mean (SD)]	85.7 (8.8)	82.5 (7.7)	86.0 (6.2)	84.6 (7.4)	0.096
Stenosis length [mean (SD), mm]	6.4 (2.8)	5.9 (1.8)	7.9 (2.9)	6.8 (2.6)	0.004
Mori type (%)					0.126
A	4 (21.1)	10 (27.8)	4 (9.8)	18 (18.8)	
B	10 (52.6)	23 (63.9)	29 (70.7)	62 (64.6)	
C	5 (26.3)	3 (8.3)	8 (19.5)	16 (16.7)	

*BMI, body mass index; mRS, modified Rankin Scale; NIHSS, National Institutes of Health Stroke Scale*.

For the characteristics of the target lesions in all patients, the mean rate of stenosis was 84.6 ± 7.4%, and the mean length of the lesions was 6.8 ± 2.6 mm. The mean length of the lesions in the SES group (7.9 ± 2.9 mm) was longer than that in the BMS group (5.9 ± 1.8 mm, *p* = 0.003) and the BD group (6.4 ± 2.8 mm, *p* = 0.120). The mean rate of stenosis in the BMS group (82.5 ± 7.7%) was slightly lower than that in the SES group (86.0 ± 6.2%, *p* = 0.126) and the BD group (85.7 ± 8.8%, *p* = 1); however, the difference was not significant. The BMS group tended to have more Mori type A lesions (27.8%) and fewer Mori type C lesions (8.3%) than the other two groups (*p* = 0.126), though the difference was not significant (Table 1).

### Primary Endpoints

The 30-day stroke rate of all patients was 7.3% (7/96), and the death rate was 0. Among the seven patients who suffered stroke within 30 days, four suffered ischemic stroke, two suffered hemorrhagic stroke, and one suffered both. In detail, two patients suffered in-stent thrombosis, one patient suffered in-stent thrombosis and perforator occlusion, one patient suffered distant emboli, one patient suffered vascular perforation and distant emboli, and the other two patients suffered from intraparenchymal hemorrhage (IPH). The 30-day stroke rate was higher in the BD group [15.8% (3/19)] and SES group [9.8% (4/41)] than in the BMS group (0%) (*p* = 0.047).

Of the 96 patients, 13 (13.5%) patients suffered at least one onset of ischemic stroke in the ipsilateral ICA territory within the 1-year follow-up. There were no significant differences among the three groups (*p* = 0.165), although the rate of recurrent ipsilateral ischemic stroke within 1 year in the BMS group tended to be lower (5.6%, 2/36) than that in the BD group (21.1%, 4/19) and the SES group (17.1%, 7/41). Detailed data are presented in Table 2.

**Table 2:** Primary and secondary endpoints and other adverse events.

	**Balloon**	**Balloon-mounted**	**Self-expanding**	**Total**	***p*-value**
	**dilatation**	**stent**	**stent**		
***n***	19	36	41	96	
**Primary endpoints**					
Any stroke or death within 30 days (*n*, %)	3 (15.8)	0 (0.0)	4 (9.8)	7 (7.3)	0.047
Ipsilateral ischemic stroke within 12 months (*n*, %)	4 (21.1)	2 (5.6)	7 (17.1)	13 (13.5)	0.165
**Secondary endpoints**					
Revascularization success rate (*n*, %)	15 (78.9)	34 (94.4)	41 (100)	90 (93.8)	0.006
Restenosis rate within 12 months (*n*, %)	4 (21.1)	7 (19.4)	9 (22)	20 (20.8)	1.000
**Other events**					
Residual stenosis [%, mean (SD)]	27.4 (16.7)	15.7 (10.8)	13.5 (7.8)	17.1 (12.2)	0.000
Any ischemic stroke within 12 months (*n*, %)	4 (21.1)	2 (5.6)	8 (19.5)	14 (14.6)	0.142
Myocardial infarction within 12 months (*n*, %)	0 (0)	0 (0)	0 (0)	0 (0)	1.000

### Secondary Endpoints

The overall success rate of revascularization during surgery was 93.8% (90/96) and was significantly higher in the SES group (100%) than in the BD group (78.9%) (*p* = 0.006). The mean degree of residual stenosis was 17.1 ± 12.2%, which was significantly higher in the BD group (27.4 ± 16.7%) than in the BMS group (15.7 ± 10.8%, *p* = 0.001) and the SES group (13.5 ± 7.8%, *p* = 0.000). During follow-up, the restenosis rate of all patients within 1 year was 20.8% (20/96), and there were no significant differences among the three groups. Fourteen patients (14.6%) suffered ischemic stroke within 1 year, and no patient suffered myocardial infarction; there were no significant differences among the three groups.

### Factors Associated With Primary and Secondary Endpoints

Univariate analyses showed that the strategy of endovascular treatments was associated with the stroke/death rate within 30 days (*p* = 0.047) and the revascularization success rate (*p* = 0.006). The patients with Mori type C target lesions were more likely to suffer stroke or death within 30 days (25%, 4/16) than patients with Mori type A lesions (0%, 0/18) and Mori type B lesions (4.8%, 3/62) (*p* = 0.020). Multivariate analyses showed that the restenosis rate within 1 year was also higher in Mori type C lesions (31.3%, 5/16) than in Mori type A lesions (5.6%, 1/18) (*p* = 0.035). Factors including the original stenosis rate, length of the target lesion, history of hypertension, diabetes mellitus, smoking, coronary disease, and atrial fibrillation were not significantly associated with the primary and secondary endpoints. Detailed data are presented in Table 3.

**Table 3:** Factors associated with primary and secondary endpoints.

**Factor**	***p*****-value**			
	**Stroke/death**	**Ipsilateral ischemic**	**Revascularization**	**Restenosis rate**
	**within 30 days**	**stroke within 12 months**	**success rate**	**within 12 months**
Endovascular options (BD, BMS, SES)	0.047	0.165	0.006	1.000
Mori classification (Mori A, B, C)	0.020	0.303	0.309	0.138
Smoking	1	0.065	1	0.495
Stenosis rate	0.094	0.720	0.229	0.796
Stenosis length	0.844	0.635	0.406	0.365

*BD, balloon dilatation; BMS, balloon-mounted stent; SES, self-expanding stent*.

## Discussion

In this study, we retrospectively investigated the safety and efficacy of tailored endovascular treatments for severe atherosclerotic stenosis of intracranial ICA. This was the first study with a 1-year outcome of endovascular treatment specifically for severe intracranial ICA stenosis, and it had a larger sample than prior studies. Our study revealed that the 30-day stroke rate of patients with severe intracranial ICA atherosclerotic stenosis was 7.3 and 13.5% patients suffered at least one onset of ischemic stroke in the ipsilateral ICA territory within the 1-year follow-up.

Since the SAMMPRIS and VISSIT trials, the safety and efficacy of endovascular treatment for intracranial atherosclerotic stenosis has been questioned given the negative results. The SAMMPRIS trial showed a higher rate of 30-day stroke and death in the stenting arm with the Wingspan stent (14.7%) relative to aggressive medical management (5.8%) and a higher rate of 1-year stroke and death (19.7%) relative to the medical arm (12.6%) (7). The VISSIT trial demonstrated an even higher 30-day stroke/hard TIA rate in the stenting group with a BMS (24.1%) than in the medical group (9.4%) and a much higher 1-year stroke/hard TIA rate in the stenting group (36.2%) than in the medical group (15.1%) (5). However, owing to the limitations of the trials, researchers and clinicians still regard angioplasty as a promising measure for stroke prevention in patients with severe ICAS. Researchers have begun to study the outcomes of endovascular treatments for atherosclerotic stenosis in some specific sites of intracranial arteries (18, 19). In addition, several measures have been taken to modify the endovascular treatment strategies.

Endovascular treatment for stenosis of the intracranial ICA presents a particular challenge because of the artery's location and tortuous route. Compared with other sites of intracranial arteries, the intracranial ICA is more tortuous and thus it is difficult to transport endovascular devices in place (11). Studies in endovascular therapy specific for intracranial ICA remain particularly scarce. Detailed analysis of periprocedural strokes in patients undergoing intracranial stenting in SAMMPRIS showed that 15% of patients out of 40 patients with intracranial ICA stenosis had periprocedural strokes with Wingspan stents, including 3 (7.5%) hemorrhagic events and 3 (7.5%) ischemic events. Turk et al. disfavored interventional stenting with stenosis lesions in the ICA supraclinoid segment owing to high rates of in-stent restenosis and recurrent ischemic stroke (20). However, Wang's study showed that only one patient (1.7%) out of 36 patients with atherosclerotic stenosis in the intracranial ICA treated with Wingspan stents had ischemic stroke during the 30-day perioperative period, five patients (13.9%) had ipsilateral stroke and three patients (8.3%) had ipsilateral TIA during a 6–68-month follow-up (11). Wang's study also revealed that the outcomes of patients with intracranial ICA atherosclerotic stenosis treated with endovascular therapy were acceptable; however, this finding remains to be further verified owing to the limited number of patients enrolled. In our study, a larger number of patients with severe atherosclerotic stenosis of the intracranial ICA (70–99%) were managed with endovascular treatment and were followed up for at least 1 year, and both 30-day and 1-year outcomes were shown to be acceptable.

In addition, since the SAMMPRIS trial several measures have been proposed to modify the endovascular treatment strategy, these were utilized in our study. First, we restricted the inclusion criteria. The patients selected in our study had ipsilateral hypoperfusion and poor collateral circulations from CT perfusion examination, as patients with hypoperfusion symptoms and poor collaterals were most likely to fail medical therapy and benefit from revascularization (14). Another measure taken in our study was variable device selection in cases of different lesions, as the devices exclusively used in prior studies (the Wingspan system for SAMMPRIS, the Vitesse intracranial stent for VISSIT) have not been shown to be well-suited for all ICAS lesions (17, 21). Miao used tailored angioplasty and/or stenting for ICAS, and the results showed that the complication rate within 30 days was 5.3% (14). However, longer-term outcomes of tailored endovascular treatment of ICAS have never been explored. The results of our study demonstrated that the overall 30-day stroke rate was 6.5%, the death rate was 0, and the overall 1-year ipsilateral ischemic stroke rate was 13.5%, which was much lower than that in the stenting arms of SAMMPRIS and VISSIT and comparable with that in the medical arm. Considering that the medical treatments in the trials above were too aggressive to be achieved in clinical practice, at least for a Chinese population, our study showed that outcomes of tailored endovascular treatment of intracranial ICA atherosclerotic stenosis were acceptable.

Intracranial segments of the ICA are more tortuous than other intracranial arteries, and the tailored endovascular treatment strategy used in this trial could mitigate this problem well, as different devices have inherent advantages and drawbacks and are thus suitable for different target lesions. BD alone has better flexibility but greater risk of elastic recoil and dissection. BMS has a high radial force but the flexibility is limited; it has a greater risk of injury to the vessel due to excessive straightening and is difficult to pass through tortuous vessels (22). SES has a certain degree of flexibility and conforms well to the vessel wall, but the radial force is limited; thus, it is less apt to achieve good revascularization in calcified lesions. Besides, as SES placement requires two steps (first balloon dilatation and then stenting angioplasty), a longer procedural time is required. Therefore, there was preference for BMSs for straight arterial access and Mori A lesions for better radial force and shorter procedural time, balloon pre-dilatation plus SES for tortuous arterial access and Mori B or C lesions for a certain degree of flexibility and radial force, and BD alone for more tortuous arterial access for safety.

In addition, we investigated the factors associated with the primary and secondary outcomes. Our results showed that the strategy of endovascular treatments had a significant effect on the results in patients with stenosis of the intracranial ICA. The 30-day stroke or death rate was significantly higher in the BD and SES groups than in the BMS group (0%), and the revascularization success rate was significantly higher in the SES group (100%) than in the BD group (78.9%). This was reasonable given the principle of stenting selection and the characteristics of the different endovascular strategies mentioned above. However, due to the high incidence of complications and stroke, and the low recanalization rate in BD group, it is suggested that patients with difficult lesions should be left alone, only be given medical therapy instead of intravascular therapy. Another notable and important factor was Mori type. In our study, patients with Mori type C target lesions were more likely to suffer stroke within 30 days and restenosis within 1 year. A number of papers have shown that lesions with different characteristics classified by Mori carry different risks during intracranial endovascular revascularization (17, 18). However, few studies have clearly demonstrated the incidence of primary and secondary endpoints. Mori's study showed that in type C lesions, the clinical success rate of angioplasty was 33%, the restenosis rate at 1 year was 100%, and the cumulative risk of ipsilateral ischemic stroke at 1 year was 56% (23). Our study showed that in type C lesions, the clinical success rate was 75%, the revascularization success rate was 87.5%, the restenosis rate at 1 year was 31.3%, and the annual ipsilateral ischemic rate was 25%; these results were all greatly improved when compared with Mori's. In addition to the development of techniques and richer experience with neurointerventionists, we believe modifications in our study such as tailored angioplasty also contribute much to this improvement. Even so, our study showed that both the safety and efficacy of endovascular treatments in type C lesions were still not satisfactory and more research should be performed on angioplasty for Mori type C lesions.

We also performed detailed analyses of periprocedural strokes in our study. Among the seven patients who suffered periprocedural strokes, there were six ischemic stroke events in five patients. Traditionally, ischemic strokes are categorized as perforator territory, distal embolic, or delayed in-stent thrombosis. Contrary with SAMMPRIS, in which most of the patients with ischemic stroke were perforator territories, only one patient suffered perforator occlusion in our study, while three suffered in-stent thrombosis and the other two were distal embolic strokes (24). This may be because the intracranial ICA has fewer tenuous perforators and thus fewer complications caused by perforator occlusion. Hemorrhagic strokes were categorized as subarachnoid or intraparenchymal. In our study, three patients suffered hemorrhagic stroke, among whom two suffered intraparenchymal hemorrhage and the other suffered vascular perforation. The mechanism of IPH post-stenting is uncertain, and hyperperfusion or autoregulatory dysfunction may be one possible mechanism (24). The ICA is much larger than other intracranial arteries, and hyperperfusion is thought to occur more frequently after carotid stenting, which may have contributed to IPH in our study (25).

Our study also has some limitations. First, the trial was performed in a single center and the patients enrolled were only Chinese people, which was not representative. Second, the pre-procedural NIHSS scores of patients in our study were relatively low, so there may be some extent of selection bias. In addition, this was a retrospective trial and was absent of a medical arm as the control group, therefore, the evidence-based level of this study is not so high. Also, a number of stents, such as Neuroform, Enterprise, and Solitaire, originally designed for neck remodeling in the treatment of intracranial aneurysms were used off label (21, 26, 27). Finally, the number of patients in our study was not large enough even though this is the largest study of tailored endovascular treatments for atherosclerotic stenosis of intracranial ICA.

## Conclusions

Both the 30-day and 1-year outcomes of tailored angioplasty seemed to be acceptable in the treatment of symptomatic atherosclerotic stenosis of intracranial ICA. However, this needs to be confirmed by further investigation, preferably in large multicenter randomized controlled clinical trials.

## Data Availability Statement

The original contributions presented in the study are included in the article/supplementary material, further inquiries can be directed to the corresponding author/s.

## Ethics Statement

The studies involving human participants were reviewed and approved by Institutional ethics committee at Beijing Tiantan Hospital. The patients/participants provided their written informed consent to participate in this study.

## Author Contributions

HD, DM, and ZM conceived the study and made critical revisions to the manuscript. SS and YW analyzed the data and drafted the manuscript. XH acquired most of the data. NM, FG, LS, XS, and LL participated in the data collection. All authors read and approved the submitted manuscript.

## Conflict of Interest

The authors declare that the research was conducted in the absence of any commercial or financial relationships that could be construed as a potential conflict of interest.
